# Research on the Formation Conditions and Preventive Measures of Uranium Precipitates during the Service Process of Medical Isotope Production Reactors

**DOI:** 10.3390/ma17040945

**Published:** 2024-02-18

**Authors:** Yanli Zhao, Yuan Gao, Xinyue Li, Yi Le, Yang Zhang, Jie Qiu, Yong Xin

**Affiliations:** 1 Science and Technology on Reactor System Design Technology Laboratory, Nuclear Power Institute of China, Chengdu 610213, China; zyl695662724@163.com; 2 School of Energy and Power Engineering, Xi’an Jiaotong University, Xi’an 710049, China; gy1996417@stu.xjtu.edu.cn (Y.G.); lxy98120@163.com (X.L.); yy295441549@stu.xjtu.edu.cn (Y.L.); 3123303380@stu.xjtu.edu.cn (Y.Z.); qiujie1228@xjtu.edu.cn (J.Q.)

**Keywords:** medical isotope production reactor, uranyl nitrate, radiolysis products, prevention of sedimentation

## Abstract

This study focuses on the Medical Isotope Production Reactor (MIPR), an aqueous homogeneous reactor utilized for synthesizing medical isotopes like ^99^Mo. A pivotal aspect of MIPR’s functionality involves the fuel solution’s complex chemical interactions, particularly during reactor operation. These interactions result in the formation of precipitates, notably studtite and columnar uranium ore, which can impact reactor performance. The research presented here delves into the reactions between liquid fuel uranyl nitrate and key radiolytic products, employing simulation calculations complemented by experimental validation. This approach facilitates the identification of uranium precipitate types and their formation conditions under operational reactor settings. Additionally, the article explores strategies to mitigate the formation of specific uranium precipitates, thereby contributing to the efficient and stable operation of MIPR.

## 1. Introduction

Medical isotopes are essential in the field of nuclear medicine, particularly for diagnosing and treating major diseases like cardiovascular and cerebrovascular disorders, as well as malignant tumors [1,2]. Currently, the global production of medical isotopes primarily relies on reactors such as the High-Flux Isotope Reactor and the Missury University Research Reactor in the United States, the National Research Universal Nuclear Reactor in Canada, the High Flux Reactor in the Netherlands, and the High Flux Reactor in Australia. Additionally, there are some reactors for radioisotope production located in Russia and Eastern Europe [3,4]. Most of these reactors, built in the 1950s and 1960s, are now facing challenges due to aging and an imbalance between supply and demand, with expected closures between 2016 and 2030 [5,6,7].

As a highly populated country with a substantial demand for medical isotopes, China currently possesses a comparatively limited production capacity and heavily relies on imports. In the event of scarcity in the international market, a serious threat would be posed to both Chinese public health and social stability. Therefore, it is imperative for China to prioritize and advance its research in medical isotopes, aiming to achieve self-sufficiency in their production.

At present, medical isotope production worldwide primarily depends on the irradiation of enriched uranium targets within reactors. However, this method is associated with drawbacks such as complex separation processes, high costs, the significant loss of medical isotopes during long-distance transportation, the generation of large quantities of radioactive waste, and the resulting severe environmental pollution [8,9]. Consequently, researchers have actively embarked on the exploration of alternative medical isotope production technologies. In 1997, the design concept for a medical isotope production reactor utilizing uranyl nitrate solution was proposed by the Babcock & Wilcox Company [10]. The fission products are directly produced within this uranyl nitrate solution through fission reactions involving ^235^U.

Medical Isotope Production Reactors (MIPRs) are a specialized type of aqueous homogeneous reactor, typically employing uranyl nitrate or uranyl sulfate solutions as fuels. These reactors offer numerous advantages, such as enhanced safety features, simplified production technology, a reduced generation of radioactive waste, and lower construction and operational costs. MIPRs are increasingly recognized for their potential in producing crucial medical nuclides like Molybdenum-99 (^99^Mo) [11]. Nonetheless, challenges arise during the operation of MIPRs due to the nature of the solution reactor. Key components in the solution, such as H_2_O and ${NO₃}^{-}$ , are prone to undergoing dissociation upon irradiation. This process generates a variety of radiolysis products, including hydroxyl radicals (·OH), hydrogen atoms (·H), molecular hydrogen (H_2_), hydrogen peroxide (H_2_O_2_), nitrogen (N_2_), and nitrogen oxides [12].

These radiolysis products can significantly influence the reactor’s integrity by affecting vessel corrosion [13] and altering the pH of fuel solutions, thereby impacting the speciation of uranium in the solution. For example, under high pH conditions, uranyl ions may hydrolyze, forming precipitates like UO_3_·2H_2_O, also known as columnar uranium ore [14]. Additionally, in media with elevated hydrogen peroxide concentrations, uranyl ions tend to form a polymer complex, leading to uranium peroxide precipitates such as studtite (UO_2_)(O_2_)·4H_2_O [15,16，^17^]. The presence of such sediments can impede the reactor’s normal operation.

Despite the significance of these findings, research into the formation conditions of these precipitates remains limited [18]. To address this gap, this article employs a combination of simulation calculations and experimental verification to clarify the chemical transformations of uranyl nitrate fuel solutions under operational conditions. Special attention is given to elucidate the conditions and components that lead to the precipitation of uranyl ions through hydrolysis reactions and their interactions with the main radiolysis product, H_2_O_2_. By understanding these specific chemical reactions and the resulting precipitates, this study proposes strategies to prevent sediment formation, thereby providing a scientific foundation for the stable and effective operation of MIPRs.

## 2. Research Methods

To accurately evaluate the formation of precipitates in the fuel solution during the operation of MIPRs, a comprehensive approach was utilized. Uranyl nitrate solution was used as a model fuel. Furthermore, both simulation and experimental methods were employed to investigate the conditions leading to precipitation formation. The simulation aspect of the study was conducted using The Geochemist’s Workbench (GWB), an advanced simulation software GWB 12 Standard developed by the Department of Geology at the University of Illinois at Urbana, Champaign in the United States. GWB is particularly well adept at modeling reaction pathways, calculating stability phase diagrams, and assessing reaction equilibrium states. It also facilitates the quantitative analysis of the concentrations and various component conversions in the system.

In this study, GWB was employed to model the precipitation formation conditions in the uranyl nitrate solution. Experimental verification was carried out to confirm these conditions so as to complement the simulation. The precipitates produced in the experiments were comprehensively characterized to determine their type and composition. This analysis was performed using a suite of proven techniques, including Raman spectroscopy, scanning electron microscopy coupled with energy dispersive spectroscopy (SEM-EDS), and powder X-ray diffraction. These techniques provided detailed insights into the nature of the precipitates, thereby contributing significantly to the understanding of MIPR operation and enhancing the ability to control and optimize reactor performance.

## 3. Model Establishment and Experimental Process

### 3.1. Modeling of Precipitation Formation Conditions

The GWB Standard suite incorporates a variety of integrated program modules, among which the TEdit and Act2 modules are of particular significance. The TEdit module plays a crucial role in augmenting the existing thermodynamic database, which is named thermo.com.V8.R6.full, by incorporating supplementary thermodynamic data pertinent to uranyl peroxide complexes. Conversely, the Act2 module is important in evaluating the stability of specific species, depicting the various manifestations of these species within a solution matrix under fluctuating conditions.

The methodology for modeling and computation description is as follows:

1. Preliminary Analysis: This stage involves a detailed examination of the target species and the equilibrium constants of the fundamental reactions for the ancillary species utilized in formulating these reactions. Subsequently, the researched equilibrium constant K, as delineated in Table 1, is integrated into the thermodynamic database.
2. Configuration in Act2 Interface: At this stage, it is necessary to set the main species for computation within the Act2 interface (in this instance, UO_2_^2+^). Additionally, this stage involves the adjustment of two principal variables that influence the morphology of the key species—specifically, the pH value and hydrogen peroxide activity for this process. This step culminates with the definition of the overall solution system, including establishing the initial concentration of other species within the system.
3. Computational Process in Act2: At this stage, Act2 computes the initial equilibrium state of the reaction. Subsequently, it employs the Gibbs free energy minimization method to deduce the unique convergent equilibrium state of the system for each specified pH value and hydrogen peroxide activity. The outcome is the simulation of a phase diagram for the key species, which vividly illustrates the diverse forms of the key species (ions, complexes, precipitates, gases, etc.) under the collective influence of multiple variables.

### 3.2. Experimental Process

Laboratory experimentation was conducted to elucidate the chemical behaviors of uranyl nitrate in the presence of varying hydrogen peroxide concentrations. The methodological framework for these experiments is delineated as follows:

#### 3.2.1. Preparation of Reaction Mixtures

An amount of 4.85 g uranium nitrate hexahydrate was dissolved in the 10 mL 0.5 mol/L nitric acid to give 0.966 mol/L stock solution. Then, 1000, 333, 111, 37, and 12 μL of 30% hydrogen peroxide were diluted to 10 mL with a certain amount of deionized water in volumetric flasks, respectively. Hydrogen peroxide solutions with a concentration of 0.979, 0.326, 0.109, 0.036, and 0.012 mol/L were prepared. Next, 82, 27, and 9 μL of 30% hydrogen peroxide was diluted to 200 mL with a certain amount of deionized water in volumetric flasks, respectively. Hydrogen peroxide solutions with concentrations of 0.004, 0.0013 and 4.47 × 10^−4^ mol/L were prepared. Then, 9 μL of 30% hydrogen peroxide was diluted to 600 mL with a certain amount of deionized water to give a hydrogen peroxide solution with a concentration of 1.49 × 10^−4^ mol/L. An aliquot of 200 μL of stock solution was introduced into a glass reaction vessel. To the stock solution, 100 μL of hydrogen peroxide solutions of diverse concentrations were added. These reactions were conducted at ambient temperature, as well as at controlled temperatures of 50 °C and 80 °C in baking oven. The resultant final concentrations of hydrogen peroxide ranged from 0.326 mol/L, 0.109 mol/L, 0.036 mol/L, 0.012 mol/L, 0.004 mol/L, 0.0013 mol/L, 4 × 10^−4^ mol/L, 1.49 × 10^−4^ mol/L, to 4.97 × 10^−5^ mol/L. Each reaction solution was maintained at the respective temperature for a duration of four hours to facilitate reaction progression while observing the resultant chemical state in detail.

#### 3.2.2. Isolation of Precipitates

The reaction mixtures with a hydrogen peroxide concentration of 0.326 mol/L, conducted at 25 °C, 50 °C, and 80 °C, a light yellow precipitate was formed. This precipitate was subsequently separated from the reaction mixture, filtered, and washed with distilled water three times. The precipitates were then distinctly recorded as 25 °C precipitate, 50 °C precipitate, and 80 °C precipitate, corresponding to the temperatures at which they were formed.

### 3.3. Characterization of Precipitates

#### 3.3.1. Field Emission Scanning Electron Microscope

Field emission scanning electron microscope (SEM) images were obtained using TESCANMAIA3 LMH (10kV), and energy dispersive spectrometer analysis was conducted by EDAX Octane Super (10kV). All samples were measured after Gold sputtering, a treatment to improve the conductivity of the sample.

#### 3.3.2. Powder X-ray Diffraction (PXRD)

PXRD data of precipitates at 25 °C, 50 °C, and 80 °C were collected using a Bruker D8 Advance diffractometer with Cu-Kα irradiation. The 2θ range was 10°–60° with a step size of 0.02°. All samples were dried at room temperature and ground evenly in a quartz mortar. Samples were placed on a quartz glass sample holder for measurement.

#### 3.3.3. Raman Spectroscopy

The Raman spectra were collected on a Renishaw inVia Qontor Raman spectrometer with 785 nm excitation. A small amount of powder was put on tinfoil, and the Raman spectrum was collected at room temperature (25 °C).

## 4. Results and Discussion

### 4.1. Simulation Calculation of Sedimentation Boundary Conditions

**Figure 1:**
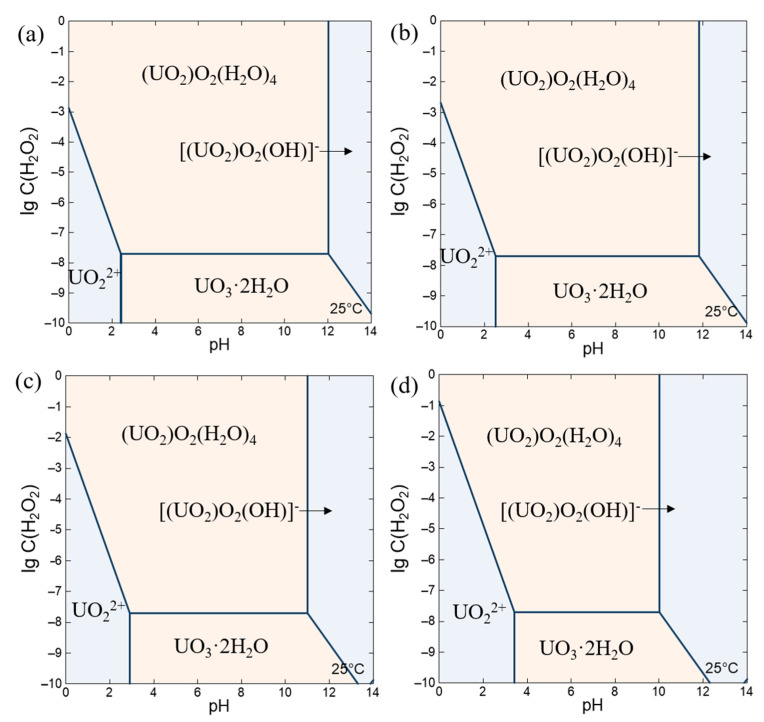
Relationship between chemical form, pH value and hydrogen peroxide concentration at different uranyl ions concentration: (**a**) 1 mol/L; (**b**) 0.64 mol/L; (**c**) 0.1 mol/L; (**d**) 0.01 mol/L.

Taking prior research into account which was undertaken at Argonne National Laboratory in the United States, it was noted that the primary determinants impacting the stability of uranyl nitrate solutions in homogeneous solution reactors are the hydrolysis of uranyl ions and the formation of precipitated species in conjunction with radiolysis products like hydrogen peroxide. Utilizing the Geochemist’s Workbench (GWB), the stability phase diagram is calculated with the concentration of 1 mol/L, 0.64 mol/L, 0.1 mol/L and 0.01 mol/L uranyl ions, taking into account variations in hydrogen peroxide concentration and pH value at 25 °C. As illustrated in Figure 1, a comprehensive visual representation of the findings is well provided.

As presented in Figure 1, the stability of uranyl ions is synergistically influenced by the concentration of hydrogen peroxide and the pH value of the solution. Within the pH range (0~14) and hydrogen peroxide concentration range (1 mol/L~1 × 10^−10^ mol/L) shown in the Figure 1, the main precipitate species formed in the solution are studtite ((UO_2_)(O_2_)·4H_2_O) and UO_3_·2H_2_O, and the main solution species are UO_2_^2+^ in low pH and uranyl peroxide hydroxyl complex [UO_2_O_2_(OH)]^−^ in high pH, respectively.

When the concentration of uranyl ions is 0.64 mol/L as an example, clarify the relationship between the uranium species, the concentration of hydrogen peroxide and pH value. Within a reaction solution where the concentration of uranyl ions is C(UO_2_^2+^) = 0.64 mol/L, the limit concentration of hydrogen peroxide is approximately 0.0021 mol/L at a pH of 0. Exceeding this concentration, the fuel solution become saturated with respect to studtite, leading to the precipitation of uranyl ions as studtite and thereby impeding the normal function of the reaction solution. Notably, an inverse correlation is observed between the pH value and the permissible maximum concentration of hydrogen peroxide for the studtite formation. This relationship is quantitatively described by the linear function: lgC(H_2_O_2_)_max_ = −1.9996 lgC(H^+^) − 2.676.

A pivotal transition occurs when the pH value reaches 2.514, marked by a change in the nature of the precipitated species within the solution. At this pH level, the dominant precipitate species transitions to studtite when the hydrogen peroxide concentration exceeds 1.98 × 10^−8^ mol/L. Conversely, when the concentration of hydrogen peroxide falls below this threshold, the observed precipitate species observed is UO_3_·2H_2_O. Under these conditions, the stability of uranyl ions in the solution is primarily governed by their hydrolysis. The formation of this particular precipitate remains unaffected even with further reductions in hydrogen peroxide concentration.

As the initial concentration of uranyl ions increases, the trend of changes in solution species in the reaction system is basically the same, but the pH value slightly decreases when the precipitated species begin to form. Apart from this, it is found according to the results of experiments that the limit concentration of hydrogen peroxide to prevent the form of studtite is expected to reduce correspondingly with the increase in uranyl ion concentration at a pH of 0.

The reaction formula related to the precipitation species mentioned above is [12]:

UO_2_^2+^ + 3H_2_O(L) ⇌ UO_3_·2(H_2_O)↓ + 2H^+^ (1)

3H_2_O → ·H + ·OH + H_2_ + H_2_O_2_ + e_aq_^−^ + HO_2_· + H_3_O^+^ (2)

UO_2_^2+^ + H_2_O_2_ + 4H_2_O ⇌ (UO_2_)(O_2_)·4H_2_O↓ + 2H^+^ (3)

H_2_O_2_ → 0.5O_2_ + H_2_O (4)

### 4.2. Verification Experiment of Precipitationlimit Conditions

**Figure 2:**
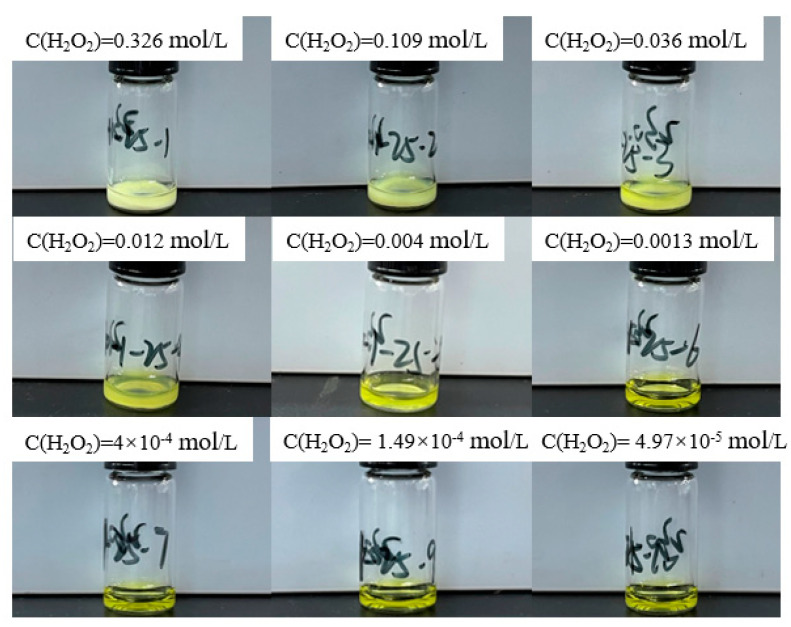
Experimental results of adding different concentrations of hydrogen peroxide solution to 0.64 mol/L uranyl ion at 25 °C.

To validate the simulation results regarding the precipitation boundary conditions in uranyl nitrate solutions, as illustrated in Figure 1, an empirical approach is adopted. This involves the addition of 100 μL of hydrogen peroxide at varying concentrations to 200 μL of a 0.966 mol/L uranyl ion solution (approximate pH of 0.64). The resultant final concentration of uranyl ions, in conjunction with different hydrogen peroxide concentrations, is maintained at 0.64 mol/L. The corresponding final concentrations of hydrogen peroxide and the experimental outcomes are systematically presented in Figure 2.

The empirical correlation between the maximum permissible concentration of hydrogen peroxide C(H_2_O_2_)_max_ and the pH value suggests that the upper limit for the isolation of studtite in a uranyl nitrate solution is 1.1 × 10^−4^ mol/L at a pH of 0.64. Within the reaction system, the hydrogen peroxide concentration spans a range of 4.97 × 10^−5^ mol/L to 0.326 mol/L. Observational analysis of the solution state reveals that at hydrogen peroxide concentrations below 1.49 × 10^−4^ mol/L, the solution remains transparent, indicating the absence of uranyl ion precipitation. Conversely, as the hydrogen peroxide concentration incrementally reaches 0.004 mol/L, the reaction solution exhibits turbidity, signifying the formation of precipitates. Thus, it is discernible that controlling the concentration of hydrogen peroxide below the simulated maximum threshold effectively inhibits precipitate formation, thereby corroborating the accuracy of the simulation results.

### 4.3. Experimental Study on the Influence of Temperature on Precipitation Formation Conditions

**Figure 3:**
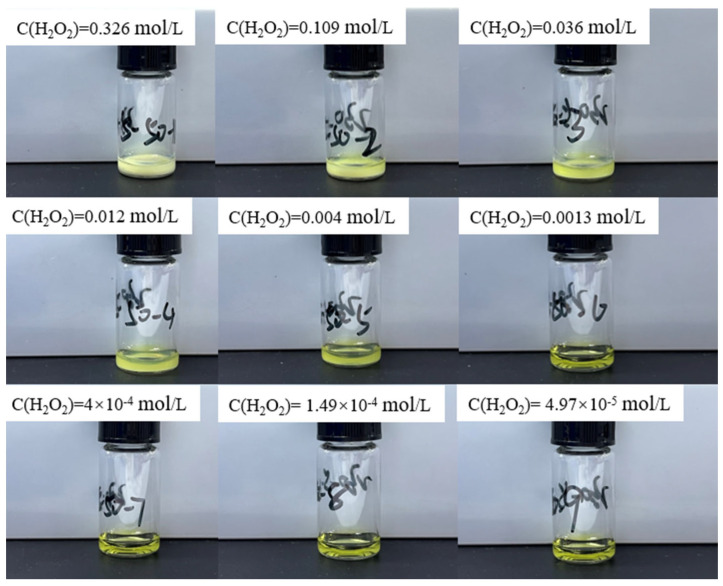
Experimental results of adding different concentrations of hydrogen peroxide solution to 0.64 mol/L uranyl ion at 50 °C.

**Figure 4:**
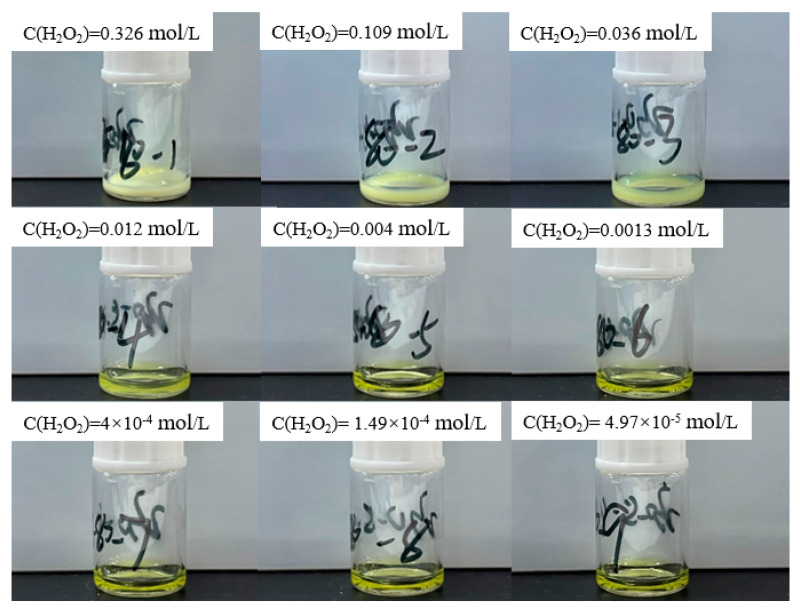
Experimental results of adding different concentrations of hydrogen peroxide solution to 0.64 mol/L uranyl ion at 80 °C.

Current limitations in obtaining thermodynamic parameters of uranyl peroxide species at varying temperatures preclude the use of GWB software for calculating the boundary conditions for precipitate formation at temperatures other than ambient. Consequently, supplementary experimental investigations are warranted. To this end, 100 μL of hydrogen peroxide at varying concentrations is added to 200 μL of a 0.966 mol/L uranyl ion solution (approximate pH of 0.64) under temperatures of 50 °C and 80 °C, respectively. The final concentration of uranyl ions in conjunction with different hydrogen peroxide concentrations is maintained at 0.64 mol/L. The corresponding concentrations of hydrogen peroxide and the experimental outcomes are systematically presented in Figure 3 and Figure 4.

At a reaction temperature of 50 °C, the compositional state of precipitate formation in the reaction solution mirrors that observed at 25 °C: the solution remains clear with no precipitate formation at hydrogen peroxide concentrations below 1.49 × 10^−4^ mol/L. However, upon reaching a concentration of 0.004 mol/L, the solution transforms into a suspension, indicating precipitate formation. At 80 °C, the reaction solution retains clarity even when the hydrogen peroxide concentration is 0.004 mol/L, with precipitate formation only occurring when the concentration increases to 0.012 mol/L. This phenomenon is attributed to the propensity of hydrogen peroxide to undergo thermal decomposition at 80 °C, leading to a reduction in its effective concentration available for reacting with uranyl ions. Consequently, the maximum hydrogen peroxide concentration corresponding to precipitate appearance in the solution increases accordingly at higher temperatures.

### 4.4. Characterization of Precipitated Species

To elucidate the microstructural attributes and phase composition of the precipitates formed in the experimental solution, a comprehensive analytical approach employing Raman spectroscopy, scanning electron microscopy (SEM) coupled with energy dispersive spectroscopy (EDS), and powder X-ray diffraction (PXRD) was implemented. These techniques were applied to characterize the precipitates synthesized at temperatures of 25 °C, 50 °C, and 80 °C.

**Figure 5:**
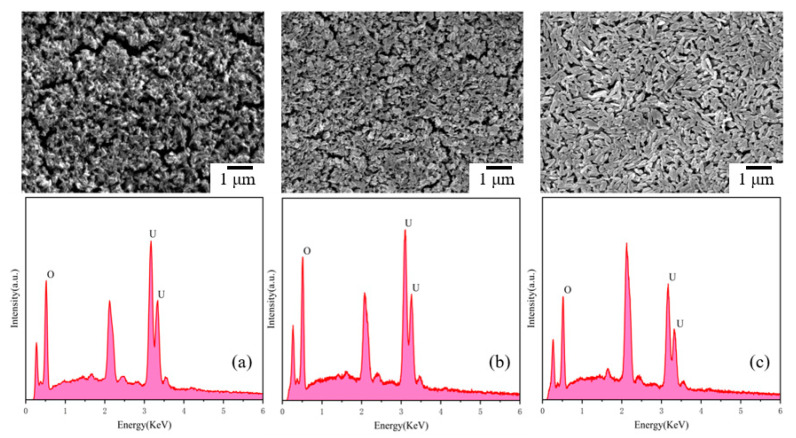
Scanning electron microscopy and energy spectrum of precipitate species generated at different reaction temperatures: (**a**) 25 °C; (**b**) 50 °C; (**c**) 80 °C.

Figure 5, depicting the SEM images at a 30,000× magnification, reveals significant disparities in the particle size and morphology of the sediments formed at 25 °C and 50 °C. Predominantly, these particles are uneven and irregular, with a size distribution centered around 0.2 μm. In contrast, at 80 °C, the precipitate comprises uniformly larger cylindrical microcrystals. The EDS analysis corroborates the predominance of Uranium (U) and Oxygen (O) as the primary constituents of the powder. The results of EDS analysis is summarized in Table 2. Considering that EDS data are mainly used to qualitatively and semi-quantitatively analyze the elements composition of solid phases, it is inaccurate to predict the certain ratio of U and O further identify specific composition of precipitate. Therefore, other characterization is conducted to prove the existence of studtite.

**Figure 6:**
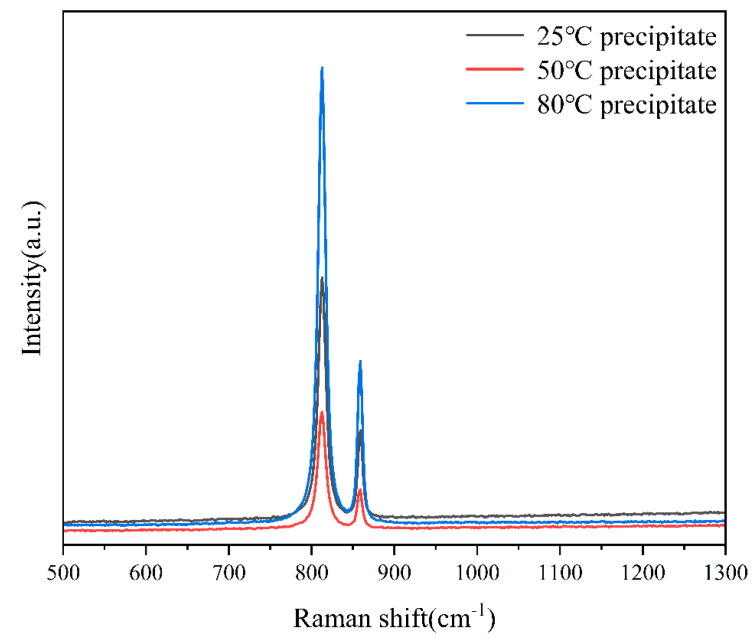
Raman spectroscopy test results of precipitate species generated at different temperatures.

In the Raman spectral analysis presented in Figure 6, two satellite peaks at 818 cm^−1^ and 864 cm^−1^ are observed in all precipitates. The peak at 818 cm^−1^ is attributable to the symmetric stretching vibration of the uranyl oxygen bond in the uranyl ion, while the peak at 864 cm^−1^ is associated with the stretching vibration of the bridging peroxy ligand. These vibrational characteristics are indicative of the chemical bonds present in studtite ((UO_2_)(O_2_)·4H_2_O) [23]. The integration of SEM-EDS and Raman data suggests the presence of studtite in the precipitate.

**Figure 7:**
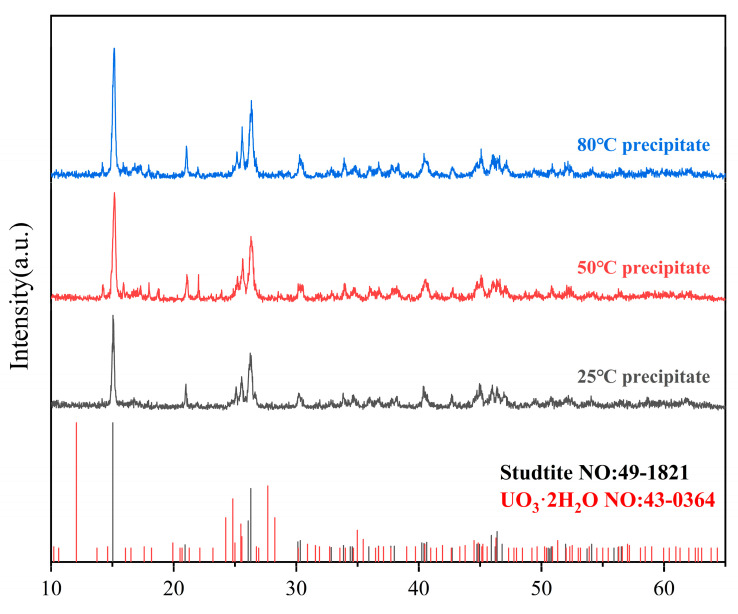
Powder X-ray diffraction patterns of precipitate species generated at different temperatures.

However, to ascertain the purity of studtite and exclude the possibility of uranium oxide existence in the precipitate, PXRD was conducted, with the results illustrated in Figure 7. The PXRD pattern exhibits characteristic peaks at 2θ values of 15.0, 20.9, 25.5, 26.1, and 26.3, which aligns closely with the standard spectrum of studtite (Powder Diffraction File n. 49-1821). The diffractograms show small additional peaks of other solid(s) phases present in amounts not exceeding 5%, which may correspond to a trace of UO_3_·2H_2_O (Powder Diffraction File n. 49-1821) or other unknown uranium oxide phases.

In conclusion, the collective data from SEM-EDS, Raman spectroscopy, and PXRD conclusively demonstrate that under the experimental conditions of pH 0.64 and reaction temperatures of 25 °C, 50 °C, and 80 °C, the precipitates formed in the uranyl nitrate solution reacted with hydrogen peroxide are the main phase of studtite.

### 4.5. Precipitation Prevention Mechanism

The analysis elucidates that the behavior of UO_2_^2+^ ions in uranyl nitrate solutions is predominantly influenced by the solution’s acidity. In conditions of low acidity, uranyl ions are prone to hydrolysis, leading to the formation of UO_3_·2H_2_O precipitates. For instance, in a uranyl nitrate solution with a concentration of 0.64 mol/L, computational simulations indicate that hydrolysis of UO_2_^2+^ ions to form UO_3_·2H_2_O precipitates occurs when the pH exceeds 2.514. This hydrolysis reaction is modulated by the synergistic effects of the UO_2_^2+^ concentration and the solution’s pH [12]. Notably, this process is reversible, implying that increasing the acidity of the solution can mitigate the formation of precipitates.

However, it is critical to recognize that the formation of UO_3_·2H_2_O is contingent upon extremely low concentrations of hydrogen peroxide. In nuclear fuel solutions, water is susceptible to radiolytic decomposition, generating hydrogen peroxide, which significantly influences the stability of the fuel. The primary precipitate affecting fuel stability during reactor operation involves the interaction between uranyl ions and hydrogen peroxide, leading to the formation of studtite. This reaction can transpire across a broad pH spectrum and a wide range of hydrogen peroxide concentrations. Elevated operational temperatures can potentially inhibit this reaction by accelerating the decomposition of hydrogen peroxide.

Operational strategies to prevent the accumulation and subsequent precipitation of studtite in the solution include reducing the pH of the solution, increasing the operating temperature, and lowering the concentration of hydrogen peroxide. These measures are essential for maintaining the stability of the fuel solution and ensuring efficient reactor operation.

## 5. Conclusions

In this scholarly article, a comprehensive study is conducted using The GWB simulation calculations in conjunction with experimental verification to investigate the interaction between uranyl nitrate and radiolysis products. The research focuses on identifying the types and formation conditions of uranium precipitates under typical reactor operating conditions. This investigation is pivotal in proposing effective strategies to prevent the formation of specific uranium precipitates, thereby offering essential theoretical support for the stable operation of nuclear reactors like MIPR. The key conclusions of this research are as follows:

(1) Simulation Analysis with GWB**: Employing GWB software, the study simulates the evolution of species in a uranyl nitrate solution at 25 °C, with a uranyl ion concentration of 1 mol/L, 0.64 mol/L, 0.1 mol/L and 0.01 mol/L across different pH values and hydrogen peroxide concentrations. This model meticulously considers the fundamental reactions of the species and their equilibrium constants. The simulation results are corroborated by similar findings reported in the scientific literature.
(2) Model Insights and pH Management**: According to the model outcomes, for a uranyl ion concentration of 0.64 mol/L, it is feasible to prevent the formation of hydrolysis-related precipitates by maintaining the pH of the solution below 2.514. Additionally, when the pH is below 2.514, to avert the precipitation of uranium peroxide in the solution, the concentration of hydrogen peroxide must be controlled to remain below the critical concentration limit corresponding to the specific pH value.
(3) Experimental Verification and Precipitate Characterization**: Through experimental approaches involving the addition of varied concentrations of hydrogen peroxide to a uranyl nitrate solution, the boundary conditions for the precipitation of uranyl ions as studtite were validated. Advanced analytical techniques, including Raman spectroscopy, SEM-EDS, and PXRD, were utilized to confirm that the precipitates are indeed composed of pure phase studtite.
(4) Proposed Measures to Prevent Precipitation**: Building on the understanding of the type of precipitation and its formation conditions, the study proposes specific measures to inhibit the formation of uranium precipitates. To suppress the formation and accumulation of UO_3_·2H_2_O, it is necessary to maintain high solution acidity, specifically controlling the pH to be less than 2.514 for a 0.64 mol/L uranyl nitrate solution. To prevent the formation and accumulation of studtite, controlling the pH and increasing the operating temperature are recommended.

## Figures and Tables

**Table 1:** Supplementary related reactions and thermodynamic data in GWB thermodynamic database [19,20,21,22].

Reaction Equation	$\log_{10}K$
$UO_{2}^{2+}+HO_{2}^{-}+H_{2}O\left(l\right)={\left[UO_{2}\left(O_{2}\right)\left(OH\right)\right]}^{-}+2H^{+}$	−(2.340 ± 0.070)
$2UO_{2}^{2+}+2HO_{2}^{-}+H_{2}O\left(l\right)={\left[{\left(UO_{2}\right)}_{2}{\left(O_{2}\right)}_{2}\left(OH\right)\right]}^{-}+3H^{+}$	7.990 ± 0.160
$UO_{2}^{2+}+H_{2}O_{2}\left(l\right)+4OH^{-}={\left[UO_{2}\left(O_{2}\right){\left(\mathrm{OH}\right)}_{2}\right]}^{2-}+2H_{2}O\left(l\right)$	28.7 ± 0.4
$UO_{2}^{2+}+2H_{2}O_{2}\left(l\right)+6OH^{-}={\left[UO_{2}{\left(O_{2}\right)}_{2}{\left(\mathrm{OH}\right)}_{2}\right]}^{4-}+4H_{2}O\left(l\right)$	36.8 ± 0.2
$(UO_{2})O_{2}\cdot 4(H_{2}O)\left(s\right)⇌UO_{2}^{2+}+H_{2}O_{2}(\mathrm{aq})+4H_{2}O\left(l\right)$	−2.87

**Table 2:** The mass percentage and atomic percentage of uranium and oxygen in 25 °C, 50 °C and 80 °C precipitate determined by EDS.

	Elements	Weight %	Atomic %
25 °C precipitate	O	18.75	77.45
	U	81.25	22.55
50 °C precipitate	O	13.93	70.66
	U	86.07	29.34
80 °C precipitate	O	23.47	82.03
	U	76.53	17.97

## Data Availability

The raw data generated during the present study are available from the corresponding author on reasonable request.

## References

[1] Wang Y., Guo Z., Wang L., Li J., Jiao L., Gao X., Weng H., Lin M. (2022). Development Status and Prospects of Electron Accelerator Production of Medical Isotope Molybdenum-99. Isotopes.

[2] Knapp F.F., Dash A. (2016). Introduction: Radiopharmaceuticals Play an Important Role in Both Diagnostic and Therapeutic Nuclear Medicine.

[3] Li Z., Han Y., Wang X., Zhang J., Wang Y., Huang Q. (2019). Production status and prospects of medical radioactive isotopes 99Mo/99mTc. Rev. Nucl. Phys..

[4] Ball R.M. (1997). Characteristic of Nuclear Reactors Used for the Production 99Mo.

[5] Lyra M., Charalambatou P., Roussou E., Fytros S., Baka J. (2011). Material Alternative production methods to face global molybdenum-99 supply short term. Hell. J. Nucl. Med..

[6] Gao F., Lin L., Liu Y., Ma X. (2016). The current status and technological prospects of medical isotope production. Isotopes.

[7] Wu H., Zhao H. (2020). Medical isotope supply faces transportation and distribution challenges due to the epidemic. Foreign Nuclear News.

[8] International Atomic Energy Agency (2008). Homogeneous Aqueous Solution Nuclear Reactors for the Production of Mo-99 and other Short Live Radiologists.

[9] Peng H.L., Tran H.H., Sembiring T.M., Arbie B. (2014). Conceptual design of a new homologous reactor for medical radiomotope Mo-99/Tc-99m production. AIP Conf. Proc..

[10] BALL R M (1997). Medical Isotope Production Reactor. U.S. Patent.

[11] Luo Q., Liu S. (2006). A water solution reactor for producing 99Mo, 131I, and 89Sr medical isotopes. Guangdong Trace Elements Sci..

[12] Lane J.A., Macpherson H.O., Maslan F. (1958). Fluid Fuel Reactors.

[13] Silverman L., Sallach R., Seitz R., Bradshaw W. (1958). Catalyst Decomposition of Hydrogen Peroxide and Uranyl Peroxide. Ind. Eng. Chem..

[14] Knope E.K., Soderholm L. (2013). Solution and Solid State Structural Chemistry of Actinide Hydrates and Their Hydrolysis and Condensation Products. Chem. Rev..

[15] Burns P.C., Hughes K.A. (2003). Studtite, [(UO_2_)O_2_(H_2_O)_2_](H_2_O)_2_: The first structure of a peroxide mineral. Am. Mineral.

[16] Forbes T.Z., Horan P., Devine T., Mcinnis D., Burns P.C. (2011). Alteration of dehydrated Schoepite and sludge to study, [(UO_2_)O_2_(H_2_O)_2_](H_2_O)_2_. Am. Mineral.

[17] Kubatko K.A.H., Helean K.B., Navrotsky A., Burns P.C. (2003). Stability of peroxide containing uranyl minerals. Science.

[18] Jerden J., Kropf J., Bakel A., Vandergrift G. (2009). Specification and Concentration of Metals in a Homogeneous Reactor Fuel Solution.

[19] Zanonato P.L., Di Bernardo P., Grenthe I. (2012). Chemical equilibria in the binary and ternary uranyl(VI)-hydroxide- peroxide systems. Dalton Trans..

[20] Zanonato P.L., Di Bernardo P., Grenthe I. (2014). A calorimetric study of the hydrolysis and peroxide complex formation of the uranyl(VI ) ion. Dalton Trans..

[21] Martínez-Torrents A., Meca S., Baumann N., Martí V., Giménez J., de Pablo J., Casas I. (2013). Uranium speciation studies at alkaline pH and in the presence of hydrogen peroxide using time-resolved laser-induced fluorescence spectroscopy. Polyhedron.

[22] Meca S., Martínez-Torrents A., Martí V., Giménez J., Casas I., de Pablo J. (2011). Determination of the equilibrium formation constants of two U(VI)-peroxide complexes at alkaline pH. Dalton Trans..

[23] Colmenero F., Bonales L.J., Cobos J., Timón V. (2017). Study of the thermal stability of studtite by in situ Raman spectroscopy and DFT calculations. Spectrochim. Acta Part A Mol. Biomol. Spectrosc..

