# Correction: Zhao et al. Research on the Formation Conditions and Preventive Measures of Uranium Precipitates during the Service Process of Medical Isotope Production Reactors. *Materials* 2024, *17*, 945

**DOI:** 10.3390/ma17194932

**Published:** 2024-10-09

**Authors:** Yanli Zhao, Yuan Gao, Xinyue Li, Yi Le, Yang Zhang, Jie Qiu, Yong Xin

**Affiliations:** 1 Science and Technology on Reactor System Design Technology Laboratory, Nuclear Power Institute of China, Chengdu 610213, China; zyl695662724@163.com; 2 School of Energy and Power Engineering, Xi’an Jiaotong University, Xi’an 710049, China; gy1996417@stu.xjtu.edu.cn (Y.G.); lxy98120@163.com (X.L.); yy295441549@stu.xjtu.edu.cn (Y.L.); 3123303380@stu.xjtu.edu.cn (Y.Z.); qiujie1228@xjtu.edu.cn (J.Q.)

## 1. Text Correction

There were two errors in the original publication [1]. In Abstract, the term “water filamentous uranium ore” was not professional. Instead, “studtite” is more suitable and preferred for accurate representation. 

A correction has been made to Abstract:

This study focuses on the Medical Isotope Production Reactor (MIPR), an aqueous homogeneous reactor utilized for synthesizing medical isotopes like ^99^Mo. A pivotal aspect of MIPR’s functionality involves the fuel solution’s complex chemical interactions, particularly during reactor operation. These interactions result in the formation of precipitates, notably studtite and columnar uranium ore, which can impact reactor performance. The research presented here delves into the reactions between liquid fuel uranyl nitrate and key radiolytic products, employing simulation calculations complemented by experimental validation. This approach facilitates the identification of uranium precipitate types and their formation conditions under operational reactor settings. Additionally, the article explores strategies to mitigate the formation of specific uranium precipitates, thereby contributing to the efficient and stable operation of MIPR.

In Paragraph 1 in the Section 3.2.1, “deionized water” should be “0.5 mol/L nitric acid”. 

A correction has been made to Section 3.2.1, Paragraph 1:

An amount of 4.85 g uranium nitrate hexahydrate was dissolved in the 10 mL 0.5 mol/L nitric acid to give 0.966 mol/L stock solution. Then, 1000, 333, 111, 37, and 12 μL of 30% hydrogen peroxide were diluted to 10 mL with a certain amount of deionized water in volumetric flasks, respectively. Hydrogen peroxide solutions with a concentration of 0.979, 0.326, 0.109, 0.036, and 0.012 mol/L were prepared. Next, 82, 27, and 9 μL of 30% hydrogen peroxide was diluted to 200 mL with a certain amount of deionized water in volumetric flasks, respectively. Hydrogen peroxide solutions with concentrations of 0.004, 0.0013 and 4.47 × 10^−4^ mol/L were prepared. Then, 9 μL of 30% hydrogen peroxide was diluted to 600 mL with a certain amount of deionized water to give a hydrogen peroxide solution with a concentration of 1.49 × 10^−4^ mol/L. An aliquot of 200 μL of stock solution was introduced into a glass reaction vessel. To the stock solution, 100 μL of hydrogen peroxide solutions of diverse concentrations were added. These reactions were conducted at ambient temperature, as well as at controlled temperatures of 50 °C and 80 °C in baking oven. The resultant final concentrations of hydrogen peroxide ranged from 0.326 mol/L, 0.109 mol/L, 0.036 mol/L, 0.012 mol/L, 0.004 mol/L, 0.0013 mol/L, 4 × 10^−4^ mol/L, 1.49 × 10^−4^ mol/L, to 4.97 × 10^−5^ mol/L. Each reaction solution was maintained at the respective temperature for a duration of four hours to facilitate reaction progression while observing the resultant chemical state in detail.

## 2. Error in Figure and Table

In the original publication [1], there was a mistake in Figure 3 as published. Figure 3 inadvertently included two duplicated images of reaction solutions due to the similarity among images. To rectify this issue, we have replaced the duplicated images with the correct ones. The corrected Figure 3 appears below. 

In the original publication [1], there was a mistake in Table 2 as published. Names of elements U and O were mistakenly reversed in the last two lines of the Table 2. A correction has been made. The corrected Table 2 appears below. 

The authors state that the scientific conclusions are unaffected. These corrections were approved by the Academic Editor. The original publication has also been updated.

## Figures and Tables

**Figure 3:**
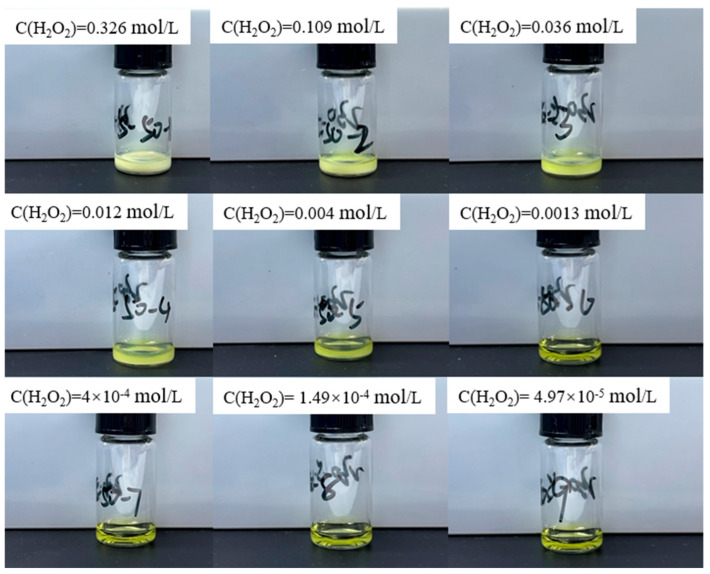
Experimental results of adding different concentrations of hydrogen peroxide solution to 0.64 mol/L uranyl ion at 50 °C.

**Table 2:** The mass percentage and atomic percentage of uranium and oxygen in 25 °C, 50 °C and 80 °C precipitate determined by EDS.

	Elements	Weight %	Atomic %
25 °C precipitate	O	18.75	77.45
	U	81.25	22.55
50 °C precipitate	O	13.93	70.66
	U	86.07	29.34
80 °C precipitate	O	23.47	82.03
	U	76.53	17.97
